# Depression and sleep disturbances in children and adolescents with familial Mediterranean fever: a prospective case-control study

**DOI:** 10.1590/1516-3180.2025.3355.13052026

**Published:** 2026-08-21

**Authors:** Şeyda Doğantan, Evin İlter Bahadur, Burcu Bozkaya Yücel, Esma Bekece

**Affiliations:** I Pediatric Rheumatology Specialist, Department of Pediatric Rheumatology, Başakşehir Çam and Sakura City Hospital, Istanbul, Türkiye; PhD Candidate, Institute of Graduate Studies in Health Sciences, Istanbul University, Istanbul, Türkiye.; II Medical Doctor, Department of Developmental Pediatrics, Mersin City Hospital, Mersin, Türkiye.; III Pediatric Rheumatology Specialist, Department of Pediatric Rheumatology, Samsun Research and Education Hospital, Samsun, Türkiye.; IV Medical Doctor, Department of Pediatrics, Başakşehir Çam and Sakura City Hospital, Istanbul, Türkiye.

**Keywords:** Familial Mediterranean Fever, Depression, Sleep Wake Disorders, Sleep Quality, Pediatric Autoinflammation, Psychological Burden, Sleep Problems

## Abstract

**BACKGROUND:**

Familial Mediterranean fever (FMF) is a hereditary autoinflammatory disorder characterized by recurrent febrile episodes and serositis.

**OBJECTIVES:**

The aim of this study was to assess the impact of FMF on sleep quality in children and adolescents and to explore the relationship between depressive symptoms and sleep disturbances in this patient population.

**DESIGN AND SETTING:**

A prospective case-control study was conducted at a secondary hospital in Istanbul between July 2024 and January 2025.

**METHODS:**

Children and adolescents diagnosed with FMF who were followed at a pediatric rheumatology clinic were included. Sleep disturbances were assessed using the Sleep Disturbance Scale for Children (SDSC), and depressive symptoms were evaluated using the Beck Depression Inventory (BDI). Correlations between these scores were analyzed.

**RESULTS:**

The mean age of the patient group (n = 97) was significantly higher than that of the control group (n = 90) (10.6 ± 2.9 years vs. 9.3 ± 2.4 years; p < 0.001). The median SDSC and BDI scores were significantly higher in the patient group than in the control group (p < 0.05). Analysis of the SDSC subscales indicated that patients with FMF experienced difficulties in sleep initiation and maintenance, sleep-related breathing problems, hyperhidrosis, arousal disturbances, sleep-wake transitions, and excessive daytime somnolence (p < 0.05). No significant differences were found between patient subgroups categorized by MEFV gene mutations in total BDI scores, total SDSC scores, all SDSC subscale scores, or depression severity classes (p > 0.05). Furthermore, no significant correlations were observed between total BDI scores and total SDSC scores or any of the SDSC subscale scores (p > 0.05)..

**CONCLUSION:**

Children and adolescents with FMF had significantly higher levels of depressive symptoms and sleep disturbances than controls.

## INTRODUCTION

 Familial Mediterranean fever (FMF) is the most common hereditary autoinflammatory disease and is typically diagnosed in the first two decades of life.^1^ The disease is particularly prevalent among specific ethnic groups, including Jewish, Arabic, Armenian, and Turkish populations.^2,3^ FMF can significantly impair physical and psychosocial well-being, especially in cases of poorly controlled or refractory disease.^1^

 Sleep is fundamental to maintaining quality of life and physical and mental health.1 The relationships between chronic inflammatory conditions, mood, and sleep disturbances are well established.^1,4^ Several studies have demonstrated that higher disease activity in FMF is negatively associated with sleep quality in children and adolescents.^1,4^ Furthermore, anxiety and depressive symptoms in children and adolescents with FMF have been strongly linked to sleep disturbances, underscoring the importance of addressing psychopathology in this population.^2,5^

 However, the complex relationship between chronic inflammation and sleep problems remains controversial, particularly regarding cause-and-effect dynamics.^2,6^

## OBJECTIVE

 In view of the foregoing, this study was conducted to assess the impact of FMF on sleep quality in children and adolescents and to explore the relationship between depressive symptoms and sleep disturbances in this patient population. 

## METHODS

### Study design and population

 This prospective, single-center, case-control study was conducted at the Pediatric Rheumatology Department of Başakşehir Çam and Sakura City Hospital, a secondary care hospital, in Istanbul, Turkey, between July 2024 and January 2025. The study protocol was approved by the Ethics Committee of Başakşehir Çam and Sakura City Hospital (KAEK 11/29.05.2024.14). The study was conducted in accordance with the principles of the Declaration of Helsinki. Written informed consent was obtained from the parents or legal guardians of all participating children and adolescents. 

### Data collection and definitions

 The study population consisted of consecutive children and adolescents diagnosed with and followed up for FMF. FMF diagnoses were made based on the Tel Hashomer/Turkish Pediatric FMF criteria.^7 -9^ The inclusion criteria were an age between 2 and 18 years and treatment with colchicine or a combination of colchicine and an interleukin-1 inhibitor for at least 6 months. The exclusion criteria were chronic comorbidities and inability to complete the questionnaires. The sample size calculation was performed using G*Power 3.1.9.6 software. The required sample size was determined based on a comparison of the mean total BDI scores between two independent groups, the patient and control groups. Assuming a moderate effect size (Cohen’s d = 0.5), α = 0.05, and 80% power, the minimum number of participants required in each group was calculated as 64. The dropout rate was set at 15% to account for potential sample attrition. Consequently, the target sample size was set at a minimum of 74 participants per group to ensure adequate statistical power and robustness of the study findings. Ultimately, the patient group consisted of 97 patients with FMF who met the study’s inclusion criteria. To ensure adequate comparability, the number of participants in the control group was planned to be at least half the number of patients with FMF. Therefore, 90 children and adolescents, matched by sex with the patient group, were randomly selected from those who presented to the outpatient clinic with complaints of extremity pain of unknown etiology. 

### Data collection

 The participants’ demographic characteristics (age and sex) and *MEFV* gene mutations were retrieved from their medical records and recorded, along with their BDI and SDSC scores, in a predesigned worksheet. 

### Beck Depression Inventory (BDI)

 The 21-item BDI was used to assess participants’ behavioral symptoms and the severity of depressive symptoms.^6^ Each item is assigned a score of zero to three points. Therefore, the highest score that can be obtained from the BDI is 63 points. BDI scores in the ranges of 0–9, 10–16, 17–29, and 30–63 points indicate none/minimal, mild, moderate, and severe depression, respectively. Validity studies of the Turkish version of the BDI were conducted by Hisli Şahin et al.^10^

### Sleep Disturbance Scale for Children (SDSC)

 Participants’ sleep disturbances were assessed using the SDSC, which has been validated for use in children aged 6–16 years.^11-14^ The 26-item, 5-point Likert-type SDSC evaluates sleep over the past six months. Each item is assigned a score of 1 to 5 points. Therefore, the total score that can be obtained from the SDSC ranges from 26 to 130 points. The SDSC includes six subscales concerning the types of common sleep disturbances: disorders of initiating and maintaining sleep (DIMS), sleep breathing disorders (SBD), disorders of arousal (DA), sleep-wake transition disorders (SWTD), disorders of excessive somnolence (DOES), and sleep hyperhidrosis (HDY). A total score of ≥ 40 indicates sleep disturbance.^11 ,12^ The validity studies of the Turkish version of the SDSC were conducted by Ağca et al.^15^

### Statistical analysis

 Statistical analyses were performed using Jamovi Project 2.3.28 (Jamovi, version 2.3.28.0, 2023, retrieved from https://www.jamovi.org) and JASP 0.19.2 (Jeffreys’ Amazing Statistics Program, version 0.19.2, 2024, retrieved from  https://jaspstats.org) software packages. Probability (p) values of ≤ 0.05 were deemed to indicate statistical significance. The results of the statistical analyses were expressed using descriptive statistics, including mean ± standard deviation values for continuous variables determined to conform to a normal distribution, median and minimum and maximum values for continuous variables determined not to conform to a normal distribution, and numbers (n) and percentage (%) values for categorical variables. The normal distribution characteristics of numerical variables were analyzed using the Shapiro–Wilk test, and the homogeneity of variances between the study groups was assessed using Levene’s test. To compare differences in numerical variables between two independent groups, the independent samples t-test was used for numerical variables determined to conform to a normal distribution, and the Mann–Whitney U test was used for numerical variables determined not to conform to a normal distribution. 

 Additionally, the chi-square test was used to compare differences in categorical variables between the study groups. In cases where the expected frequencies were < 5 in > 20% of the cells, the Fisher–Freeman–Halton test was used. The patients’ MEFV gene mutation and zygosity statuses were determined by calculating the percentages of patients with positive test results for each mutation and compound heterozygosity. 

 The Kruskal–Wallis test was used to compare MEFV mutation status groups (homozygous, heterozygous, and compound heterozygous groups) in terms of depression and sleep parameters for continuous variables. To compare categorical variables between depression severity groups, the Fisher–Freeman–Halton test was used in cases where the expected frequencies were < 5% in > 20% of the cells, and Fisher’s exact test was used for 2 × 2 contingency tables. The relationships between BDI scores and sleep parameters were assessed using Spearman’s correlation coefficients (ρ), given that these parameters did not meet the normality assumptions. 

## RESULTS

 The mean age of the patient group was significantly higher than that of the control group (10.6 ± 2.9 years vs. 9.3 ± 2.4 years; p < 0.001). No significant difference in terms of sex distribution was observed between the groups (p = 0.999). 

 Analysis of *MEFV* gene mutations revealed that M694V was the predominant pathogenic variant (63.5%), comprising 60.7% heterozygous (n = 37) and 39.3% homozygous (n = 24) genotypes, followed by the M680I mutation (18.6%), comprising 77.8% heterozygous (n = 14) and 22.2% homozygous genotypes. The distribution of patients according to their *MEFV* mutation status revealed that the most common *MEFV* mutation status was heterozygosity, seen in 42 (43.3%) patients, followed by homozygosity (28 [28.9 %]), and compound heterozygosity (27 [27.8 %]). 

 The median total BDI score (15 [6–28] vs. 10 [0–17]) and total SDSC score (73 [46–94] vs. 36 [31–50]) were significantly higher in the patient group than in the control group (p < 0.001 for both) (**Table 1**). The proportion of children with poor sleep quality (SDSC score ≥ 40) was significantly higher in the patient group than in the control group (100% vs. 22.2%; p < 0.001). Similarly, all SDSC subscale scores were significantly higher in the patient group (p < 0.001 for all subscales). The distribution of depression severity categories showed significant differences between groups, with a higher proportion of moderate depression in the FMF group (37.1% vs. 1.1%) and a lower proportion of none/minimal depression (10.3% vs. 48.9%) compared with the control group (p < 0.001) (**Table 1**). 

**Table 1:** Comparison of depression and sleep disturbance scores between groups

**Parameters**		**Control Group (n = 90)**	**FMF Group (n = 97)**	**p values**
Beck Depression Inventory^†^		10 [0–17]	15 [6–28]	< 0.001^**^
Depression severity categories^‡^				
	*None/minimal*	44 (48.9)	10 (10.3)	
	*Mild*	45 (50)	51 (52.6)	< 0.001^***^
	*Moderate*	1 (1.1)	36 (37.1)	
Total SDSC Score^†^		36 [31–50]	73 [46–94]	< 0.001^**^
Poor sleep quality (SDSC score ≥ 40)		20 (22.2)	97 (100)	< 0.001^**^
Subscales^†^				
	*DIMS*	10 [7–15]	21 [10–30]	< 0.001^**^
	*SBD*	5 [3–10]	8 [4–15]	< 0.001^**^
	*DA*	5 [3–7]	7 [3–11]	< 0.001^**^
	*SWTD*	7 [6–10]	18 [6–25]	< 0.001^**^
	*DOES*	7 [5–10]	14 [5–22]	< 0.001^**^
	*SHY*	3 [2–6]	5 [2–8]	< 0.001^**^

† Data are presented as median (minimum–maximum) for non-normally distributed variables.

‡ n (%).

* Independent samples t-test.

** Mann–Whitney U test.

*** Fisher Freeman Halton test. Bold p values indicate statistical significance (p < 0.05).

FMF, Familial Mediterranean fever; SDSC, Sleep Disturbance Scale for Children; DIMS, Disorders of Initiating and Maintaining Sleep; SBD, Sleep Breathing Disorders; DA, Disorders of Arousal; SWTD, Sleep-Wake Transition Disorders; DOES, Disorders of Excessive Somnolence; SHY, Sleep Hyperhidrosis.

 No significant differences were found between the patient subgroups created according to *MEFV* mutation status in terms of total BDI scores, total SDSC scores, all SDSC subscale scores, or depression severity classes (p > 0.05) (**Table 2**). When examining the correlation between BDI scores and sleep parameters, no significant correlations were found between the total BDI score and total SDSC score (ρ = 0.01, p = 0.925) or any of the SDSC subscale scores (**Table 3**). 

**Table 2:** Impact of MEFV mutation status on depression and sleep parameters in children and adolescents with FMF (n = 97)

**Parameters**		**Homozygous (n = 28)**	**Heterozygous (n = 42)**	**Compound heterozygous (n = 27)**	**p values**
Beck Depression Inventory^†^		15 [8–28]	15 [6–27]	16 [8–28]	0.780
Depression severity categories^‡^					
	*None/minimal*	3 (10.7)	3 (7.1)	4 (14.8)	
	*Mild*	14 (50)	24 (57.1)	13 (48.1)	0.851
	*Moderate*	11 (39.3)	15 (35.7)	10 (37)	
Total sleep score^†^		75 [46–90]	73.5 [47–94]	70 [47–93]	0.469
Sleep disturbance subscales^†^					
	*DIMS*	22.5 [10–30]	22 [12–30]	21 [13–28]	0.168
	*SBD*	8 [4–12]	8.5 [4–13]	8 [4–15]	0.376
	*DA*	8 [4–11]	7 [3–10]	7 [3–11]	0.198
	*SWTD*	17 [6–25]	18 [7–25]	18 [10–22]	0.572
	*DOES*	14.5 [7–20]	14 [5–22]	13 [6–21]	0.350
	*SHY*	4.5 [3–7]	5 [2–8]	5 [3–8]	0.565

† Data presented as median [minimum–maximum].

‡ n (%)

Abbreviations: FMF, familial Mediterranean fever; DIMS, disorders of initiating and maintaining sleep; SBD, sleep breathing disorders; DA, disorders of arousal; SWTD, sleep-wake transition disorders; DOES, disorders of excessive somnolence; SHY, sleep hyperhidrosis; NA, not applicable. Note: Kruskal–Wallis test was used for continuous variables and the Fisher Freeman Halton test for categorical variables.

**Table 3:** Correlation analysis between Beck Depression Inventory and sleep disturbance scores in children and adolescents with FMF (n = 97)

**Sleep disturbance parameters**		**Spearman’s ρ**	**p value**
Total sleep score		0.01	0.925
Sleep disturbance subscales				
	*DIMS*	−0.042	0.685
	*SBD*	0.071	0.491
	*DA*	0.022	0.833
	*SWTD*	0.111	0.281
	*DOES*	−0.031	0.762
	*SHY*	0.187	0.067

Note: Spearman’s correlation coefficient was used for analysis.Abbreviations: FMF, familial Mediterranean fever; DIMS, disorders of initiating and maintaining sleep; SBD, sleep breathing disorders; DA, disorders of arousal; SWTD, sleep-wake transition disorders; DOES, disorders of excessive somnolence; SHY, sleep hyperhidrosis.

## DISCUSSION

 Children and adolescents with FMF had significantly higher total BDI and SDSC scores than controls, indicating greater depressive symptoms and sleep disturbances. A comparative analysis of SDSC subscale scores between the patient and control groups revealed that children and adolescents with FMF are more likely to experience disorders of sleep initiation and maintenance, sleep-related breathing problems, hyperhidrosis, arousal disturbances, sleep wake transition disorders, and excessive daytime somnolence. 

 However, our analysis did not reveal significant correlations between total BDI scores and total SDSC scores or any of the SDSC subscale scores, suggesting that depressive symptoms and sleep disturbances may represent independent manifestations of FMF rather than directly related outcomes. Although this study could not establish causality among FMF, depressive symptoms, and sleep disorders, these findings suggest that both conditions may be independently associated with FMF rather than having a direct relationship with each other. 

 Sleep quality is affected by personal, societal, and environmental factors, which are reflected in individual well-being in a multidimensional way.^1^ Although FMF typically causes intermittent physical symptoms, sleep disturbance is common among patients with chronic illnesses.^1,2,4,16-19^ Incesu et al.^1^ reported a significantly higher prevalence of sleep disturbances in children and adolescents with FMF than in healthy controls. Similarly, Makay et al.^4^ reported that sleep quality was impaired in patients with FMF, mainly due to sleep delay, anxiety, night awakenings, and sleep-disordered breathing. They also demonstrated a significant positive correlation between the frequency of FMF attacks and these sleep-related impairments. In parallel, several other studies have reported significantly higher depression rates and poorer sleep quality in both pediatric and adult FMF populations.^2,3,6^

 Children and adolescents with FMF included in this study had significantly higher scores on all SDSC subscales than controls, indicating a range of sleep problems. Although the mechanisms underlying poor sleep quality in FMF remain unclear, these problems may be linked to chronic inflammation and its systemic effects. Further research is necessary to elucidate these mechanisms and develop targeted interventions to enhance the quality of life of patients with FMF. 

 Several studies have suggested that FMF-related sleep disturbances may result from inflammation and acute disease exacerbations. Acute FMF attacks can impair quality of life by causing symptoms such as difficulty falling asleep, night awakenings, and sleep-disordered breathing. Frequent FMF attacks are also negatively correlated with sleep quality.^1,2,4^ Durcan et al.^2^ specifically highlighted that FMF attacks experienced in the last month were associated with elevated depression and sleep disturbance scores. Although we did not assess the relationship between the frequency of FMF attacks and sleep quality, the elevated SDSC scores among children and adolescents with FMF included in our study suggest that FMF-related sleep disturbances are likely multifactorial, involving chronic inflammation and mood changes. 

 The interplay between sleep disturbances and mood disorders, such as depression and anxiety, in the context of various chronic diseases, including FMF, has been well documented.^20^ Celen Yoldas et al.^20^ reported that preschool-age children with chronic rheumatic diseases are more vulnerable to developmental and behavioral challenges. Similarly, in another study, sleep disturbances in children were associated with lower self-esteem, poorer academic performance, and elevated depressive symptoms.^21^ Additionally, depression has been reported to increase with disease severity. It should be noted that short-lived and self-limiting FMF attacks may explain why depressive symptoms in FMF are less severe and frequent than in other chronic diseases.^22^

 The number of participants with moderate and severe depression was significantly higher in the patient group than in the control group. Despite the higher prevalence of both depressive symptoms and sleep disturbances in the FMF group than in the controls, we did not find significant correlations between the BDI and sleep disturbance scores. This contrasts with previous findings on the relationship between these two entities. Durcan et al.^2^ did not find any significant difference in depression and anxiety scores between children with FMF and healthy control subjects. However, they noted that children with FMF who had sleep problems had higher depression and anxiety scores. Therefore, periodic assessment of sleep quality in children with chronic illnesses, such as FMF, may help assess disease progression and improve their general well-being. 

 Although homozygous, heterozygous, and compound heterozygous mutations are often associated with greater disease severity, we found that mutation status was not significantly associated with sleep disturbances or depressive symptom scores in patients with FMF, consistent with the literature.^1,2^ These findings emphasize the need for further research to clarify the role of genetic and disease-related factors in FMF-associated sleep and mood disturbances. 

 Various tools are available for evaluating sleep disturbances, including the Children’s Sleep Habits Questionnaire (CSHQ),^2,4^ the Pittsburgh Sleep Quality Index (PSQI),^1,3,17,19^ and the CDCS.^11,23^

 Although few studies to date have assessed sleep problems in children with rheumatic diseases using the SDSC, no studies have used the SDSC to assess sleep problems in children and adolescents with FMF.^23^

 A recent study evaluating children with juvenile idiopathic arthritis (JIA), another pediatric rheumatological disease, using both the Beck Depression Inventory (BDI) and the Sleep Disturbance Scale for Children (SDSC) reported significantly higher BDI and SDSC scores in patients with JIA than in healthy controls. In that study, BDI and SDSC scores were positively correlated in the JIA group, suggesting a possible interaction between depressive symptoms and sleep disturbances. Interestingly, both BDI and SDSC scores were negatively correlated with leukocyte and neutrophil counts in patients with JIA, indicating that the relationship between psychological symptoms, sleep problems, and inflammatory parameters may be complex and disease-specific. In contrast, the present study found higher BDI and SDSC scores in children and adolescents with FMF than in controls, but no significant correlation was observed between BDI and SDSC scores. This difference may reflect variations in disease course, inflammatory burden, attack patterns, and chronicity between FMF and JIA. While JIA represents a more persistent inflammatory rheumatological condition, FMF is characterized by recurrent, self-limited inflammatory attacks, which may explain why depression and sleep disturbances appear to be independent manifestations in our FMF cohort. These findings support the need for further comparative studies using standardized tools, such as the BDI and SDSC, across pediatric rheumatological diseases to clarify the shared and disease-specific mechanisms underlying sleep disturbances and depressive symptoms.^24^

 To our knowledge, this study is the first to assess the effect of FMF on sleep quality in children and adolescents and to investigate the relationship between depressive symptoms and sleep disturbances in this patient population. However, further studies are needed to validate the use of the SDSC in assessing sleep problems in children and adolescents with FMF. 

### Limitations of the study

 This study has certain limitations. First, the study design precluded establishing causality among FMF, sleep disturbances, and depressive symptoms. Longitudinal studies are needed to elucidate the directionality of these relationships and potential mediating or moderating factors. Second, the assessment of sleep disturbances relied solely on a subjective self-report questionnaire, the SDSC. Although the SDSC is a validated and widely used tool, objective measures, such as polysomnography or actigraphy, could provide more precise data on sleep architecture and disturbances. Finally, we did not evaluate potential confounding factors that may influence sleep quality, such as socioeconomic status, comorbidities, medication adherence, and family dynamics, which are likely to contribute to both sleep and mood disturbances in children and adolescents with FMF. 

## CONCLUSION

 The findings of this study highlight the significant impact of FMF on sleep quality and mood in children and adolescents. Children and adolescents with FMF had significantly higher BDI and SDSC scores than controls, indicating a strong relationship between FMF, sleep disturbances, and depressive symptoms. Despite the higher prevalence of both depressive symptoms and sleep problems in patients with FMF, our study did not find significant correlations between these conditions, suggesting that they may be independent manifestations of FMF rather than directly related to each other. Future longitudinal studies with larger and more diverse populations and objective sleep assessments are needed to clarify the causal relationships and underlying mechanisms. Additionally, tailored interventions targeting both sleep and mood disturbances may enhance the quality of life of children and adolescents with FMF. Periodic assessment of sleep quality and mental health in patients with FMF should be considered a critical component of routine clinical care. 

## Data Availability

The data supporting the findings of this study are available upon reasonable request from the corresponding author, Şeyda Doğantan.
